# Mapping the effects of specific radiation damage and solvent radiolysis in buffers and crystals with online UV–Vis absorption spectroscopy

**DOI:** 10.1107/S2059798326002743

**Published:** 2026-04-21

**Authors:** Jack Stubbs, Nicolas Caramello, Matthew J. Rodrigues, Sylvain Engilberge, Eric Mathieu, Samuel L. Rose, Gwyndaf Evans, Antoine Royant, Ivo Tews

**Affiliations:** a School of Biological Sciences and Institute for Life Sciences, Faculty of Environmental and Life Sciences University of Southampton SouthamptonSO17 1BJ United Kingdom; b Diamond Light Source Harwell Science and Innovation Campus DidcotOX11 0DE United Kingdom; c European Synchrotron Radiation Facility (ESRF) 71 Avenue des Martyrs, CS 40220 38043Grenoble CEDEX 9 France; d Institute for Nanostructure and Solid State Physics Universität Hamburg Center for Ultrafast Imaging, HARBOR, Building 610, Luruper Chaussee 149 22761Hamburg Germany; e Université Grenoble Alpes, CNRS, CEA, Institut de Biologie Structurale (IBS) 71 Avenue des Martyrs, CS 10090 38044Grenoble CEDEX 9 France; University of Oxford, United Kingdom

**Keywords:** UV–Vis absorption spectroscopy, specific radiation damage, radiolysis

## Abstract

Online UV–Vis absorption spectroscopy is often used during crystallographic data collection to characterize chromophores within proteins. This includes the study of electron-rich or redox-active enzyme intermediates and cofactors that are prone to specific radiation damage, necessitating suitable low-dose data-collection strategies. *In crystallo* spectroscopy enables the monitoring of X-ray-induced changes in the spectroscopic signatures of proteins and ligands; however, a comprehensive approach that also considers buffer components is required. Here, we present a mapping of X-ray-induced spectral changes in common crystallization chemicals and mixtures at cryogenic temperature, for which spectroscopic changes can be detected at doses as low as 1 kGy. A transient increase in absorption between 450 and 700 nm is frequently observed, arising from solvated electron absorption. Below 450 nm, several distinct absorption peaks were detected, for example for halides in buffers. In addition, Rayleigh scattering can lead to an increasing loss of photons from the optical path as the wavelength decreases, and thus to a significant elevation of the baseline at shorter wavelengths. In this study, we demonstrate the use of spectroscopic analyses to investigate the chromophoric enzyme intermediate I320 in vitamin B_6_ biosynthesis. In this case, X-ray-induced spectral changes were attributed to crystallization agents, cryoprotectants and light scattering, thereby excluding intrinsic alterations to the enzyme intermediate under low-dose conditions. Our study highlights the importance of monitoring spectral changes during diffraction data collection to ensure accurate interpretation of the electronic structure of chromophores.

## Abstract 2

Radiation-induced changes during X-ray crystallographic data collection can compromise concurrent spectroscopic studies. Suitable protocols to detect and identify spectral artefacts from crystallization buffers and cryoprotectants are essential for the reliable analysis of chromophoric ligands in macromolecules.

## 1. Introduction

UV–Vis absorption spectroscopy is a rapid, nondestructive technique used to study enzymes and their associated electronic transitions within active sites and cofactors (van Amerongen & van Grondelle, 1995 ▸). Many enzymes rely on redox centres and metalloorganic cofactors that are chromophoric, such as haem groups, flavins and iron–sulfur clusters. Monitoring of changes in absorption within the ultraviolet and visible regions allows the detection of structural changes, oxidation states and conformational dynamics, all of which drive catalytic function [see von Stetten *et al.* (2015 ▸) and references therein]. Changes in electron configuration due to ligand binding or redox reactions result in distinct spectroscopic shifts, allowing redox centres and metalloorganic cofactors to be directly probed. Real-time monitoring opens up the possibility of observing transient intermediates to describe the underlying kinetic mechanisms driving enzyme activity (Dworkowski *et al.*, 2015 ▸; von Stetten *et al.*, 2015 ▸).

A compounding factor when irradiating protein crystals with X-rays is radiation damage, resulting in X-ray photons ejecting electrons from atoms within macromolecules, ligands or solvent. The initiation of a cascade of free-radical chemistry leads to the generation of radical species and solvated electrons, which in turn react with the macromolecule, ligand or solvent to create a complex mixture of compounds (Garman, 2010 ▸; Ravelli & Garman, 2006 ▸). Chromophores are often redox-sensitive, and thus susceptible to X-ray-induced radiation damage. UV–Vis absorption spectroscopy helps to detect that an intermediate has formed and has not been affected by X-ray exposure, providing proof of its electronic structure. Mapping and monitoring specific radiation damage using this technique is thus essential to avoid misinterpretation (Orru *et al.*, 2011 ▸; Kekilli *et al.*, 2017 ▸; Roger *et al.*, 2024 ▸).

If the site of interest contains a chromophore, X-ray-induced damage to the compound is conveniently monitored by UV–Vis absorption spectroscopy as a function of the absorbed X-ray dose. This allows the detection of incurred damage to the chromophore arising from reactive chemical species generated by the X-ray beam. Examples include most metal ions, disulfides and many organic prosthetic groups (Owen *et al.*, 2011 ▸). The term specific radiation damage (SRD) denotes occurrence at sites particularly susceptible to reacting with the electrons and radical species generated during X-ray irradiation; in the experimental electron-density map, this manifests itself as a spurious gain or loss of difference electron density. In the order of decreasing sensitivity, significant SRD to proteins and ligands occurs, but is not limited to, the photoreduction of metal centres (Antonyuk & Hough, 2011 ▸), the cleavage of disulfide bonds (Helliwell, 1988 ▸; Ravelli & McSweeney, 2000 ▸; Weik *et al.*, 2000 ▸) and the decarboxylation of acidic residues (Ravelli & McSweeney, 2000 ▸). SRD has impacted the interpretation of several protein families, including metal-binding proteins (Hersleth & Andersson, 2011 ▸; Kwon *et al.*, 2017 ▸), proteins containing organic prosthetic groups (Borshchevskiy *et al.*, 2014 ▸) and enzyme active sites (Leiros *et al.*, 2001 ▸; Dubnovitsky *et al.*, 2005 ▸).

Schiff bases, often found as secondary ketamines or aldimines of protein lysine side chains, have been shown to be sensitive to SRD. Prominent examples are Schiff bases with prosthetic groups such as retinal in bacteriorhodopsin (Matsui *et al.*, 2002 ▸) or pyridoxal 5′-phosphate (PLP) in phosphoserine aminotransferase and alanine-glyoxylate aminotransferase (Giardina *et al.*, 2017 ▸; Dubnovitsky *et al.*, 2005 ▸). We previously resolved an imine relay mechanism via Schiff bases in vitamin B_6_ biosynthesis in the complex conversion of two carbo­hydrates and ammonia to the aromatic heterocycle PLP (Rodrigues *et al.*, 2017 ▸). Central to this mechanism is the covalent chromophoric I320 intermediate with a characteristic absorption maximum at ∼315 nm (Raschle *et al.*, 2007 ▸; Hanes *et al.*, 2008 ▸; Smith *et al.*, 2015 ▸; Robinson *et al.*, 2016 ▸; Rodrigues *et al.*, 2017 ▸, 2022 ▸). Crystal structures revealed that the I320 intermediate bound via a double Schiff base (Robinson *et al.*, 2016 ▸; Rodrigues *et al.*, 2017 ▸). We suspected SRD to occur and therefore performed a multi-crystal low-dose data collection (Rodrigues *et al.*, 2017 ▸). Indeed, drastic changes in the spectra during data collection were observed. At the time, this observation was puzzling, as no corresponding loss of electron density was observed with increasing dose (Rodrigues *et al.*, 2017 ▸). The apparent discrepancy can be resolved by adopting a more comprehensive approach, in which the chromophoric signature of Pdx1–I320 is shown to overlap with spectral changes arising from X-ray-induced changes attributed to crystallization buffer components and cryoprotectants.

Here, we present an online UV–Vis absorption analysis of buffer components for typical crystallization conditions, including varying molecular-weight polyethylene glycol (PEG) solutions, common buffers such as Tris [tris(hydroxy­methyl)aminomethane] and HEPES [4-(2-hydroxyethyl)-1-piperazineethanesulfonic acid] and widely used salts. Experiments were performed on ESRF beamline BM07-FIP2 (McCarthy *et al.*, 2025 ▸). We further examined the Morpheus crystallization screen, widely applied in cryo-crystallography, which employs multi-component formulations (Gorrec, 2009 ▸). Nearly all of these buffers exhibited pronounced spectral changes upon prolonged X-ray irradiation. Studying cryoprotectants, we report changes for glycerol, ethylene glycol, saccharides and PEG 400 stemming from solvated electrons, but less so for MPD (2-methyl-2,4-pentanediol). A better understanding of the sources of X-ray-induced changes in spectroscopic signatures in crystallization agents and cryoprotectants is essential, particularly in cases where the assessment of radiation damage to chromophores is challenging.

## 2. Methods

### 2.1. Protein purification and crystallization

#### 2.1.1. Protein purification

*Arabidopsis thaliana* Pdx1.3 (UniProt ID Q8L940; EC 4.3.3.6) was recombinantly expressed in *Escherichia coli* BL21 (DE3) cells as a C-terminally His-tagged protein (Tambasco-Studart *et al.*, 2005 ▸; Rodrigues *et al.*, 2017 ▸). Cells were grown in 1 l LB (lysogeny broth) medium at 37°C to an optical density of 0.6–0.8 at 600 nm. After adding 60 ml 25%(*w*/*v*) lactose per litre of culture to induce protein expression, the cultures were grown at 30°C for 16 h. The cells were lysed by sonication on ice and then centrifuged for 1 h at 311 400*g*. Proteins were purified from the supernatant using a 1 ml metal ion-affinity chromatography HisTrap HP column (Cytiva) equilibrated with lysis buffer [50 m*M* Tris–HCl buffered at pH 7.5 containing 500 m*M* sodium chloride, 10 m*M* imidazole and 5%(*v*/*v*) glycerol]. The columns were washed with 50 m*M* Tris–HCl buffered at pH 7.5 containing 500 m*M* sodium chloride, 50 m*M* imidazole and 5%(*v*/*v*) glycerol. Proteins were eluted with elution buffer [50 m*M* Tris–HCl buffered at pH 7.5 containing 500 m*M* sodium chloride, 500 m*M* imidazole and 5%(*v*/*v*) glycerol]. The eluent was further purified on a Superdex S200 26/600 column run at 2 ml min^−1^ (50 m*M* Tris–HCl pH 8.0, 200 m*M* KCl). Fractions containing Pdx1.3 were checked for purity by SDS-PAGE and pooled for concentration using Vivaspin 20 centrifugal concentrators (30 kDa molecular-weight cutoff; Sartorius).

#### 2.1.2. Crystallization

Pdx1.3 was crystallized at 20 mg ml^−1^ in 550 m*M* sodium citrate, 100 m*M* HEPES pH 7.0. For I320 intermediate formation, the Pdx1.3 crystals were sequentially soaked with 100 m*M* ribose 5-phosphate (R5P) for 15 min and 1 *M* ammonium sulfate for 30 min. The Pdx1.3 cryoprotectant contained 20%(*v*/*v*) glycerol in the crystallization buffer.

### 2.2. X-ray diffraction and online UV–Vis absorption spectroscopy

#### 2.2.1. Collection of UV–Vis absorption spectroscopy data from thin films

UV–Vis absorption spectra were collected at 100 K on ESRF beamline BM07-FIP2 (Caramello *et al.*, 2026 ▸). The incident white light was provided by a DH2000-BAL lamp (Ocean Optics) connected to the setup via a 200 µm optical fibre and focused at the sample position with a 4× reflective objective. The transmitted light was focused via the second reflective objective and transmitted via a 400 µm fibre to a QEPro spectrophotometer (Ocean Optics). Thin films of buffers were prepared in MiTeGen (Ithaca, New York, USA) loops from the following chemicals: 0.1, 0.25, 0.5, 1 and 2 *M* NaCl, 1 *M* sodium citrate, 100%(*v*/*v*) PEG 400, 50%(*w*/*v*) PEG 2000, 50%(*w*/*v*) PEG 4000, 50%(*w*/*v*) PEG 8000, 1 *M* HEPES pH 7.0, 1 *M* MES [2-(*N*-morpholino)ethanesulfonic acid] pH 6.5, 1 *M* Tris pH 7.8, 1 *M* CAPS [3-(cyclohexyl­amino)-1-propanesulfonic acid] pH 10, 1 *M* CHES (*N*-cyclohexyl-2-aminoethanesulfonic acid) pH 9.5, 20%(*v*/*v*) glycerol, 100%(*v*/*v*) ethylene glycol, 20% and 50% sucrose, 20% and 50% glucose, and 25% and 50%(*v*/*v*) MPD. Additionally, we tested the crystallization mixtures from the Morpheus Screen (Gorrec, 2009 ▸): Precipitant Mix 1, 2, 3 and 4, Buffer System 1, 2 and 3, additive mixtures carboxylic acids, monosaccharides, ethylene glycols, amino acids, nitrate/phosphate/sulfate and alcohols (all neat), as well as the halide additive mixture (1:10 diluted). Thin films were directly transferred into the cryo-stream. UV–Vis absorption spectra were collected for a total of 10 min whilst the goniometer remained stationary during X-ray irradiation. Spectra were typically acquired using a 500 ms integration time. In cases where the expected amplitude of the absorption peaks of interest was particularly low, the integration time was extended to 1000 ms.

#### 2.2.2. Collection of UV–Vis absorption spectroscopy data from protein crystals

Crystals of Pdx1.3 were mounted in loops and flash-cooled directly in the cryo-stream. Data were collected as for thin buffer films, with crystals completely and evenly bathed in the X-ray beam. The dimensions of the crystals were measured using the available on-axis viewing (OAV) system. Similar to the thin films, spectra were collected with 100 ms integration averaged over 1 s with the goniometer stationary.

### 2.3. Data analysis

#### 2.3.1. Dose calculations for films and crystals at BM07-FIP2 collected without rotation

BM07-FIP2 has a top-hat beam profile and a large 200 × 200 µm beam size. The flux was measured using a silicon pin diode (Owen *et al.*, 2009 ▸). Flux calculations assumed that the flux at the sample position scaled linearly with ring current.

The dose absorbed by each of the crystals used in the UV–Vis absorption spectroscopy experiment was calculated using *RADDOSE*-3*D* (Bury *et al.*, 2018 ▸). The average dose absorbed by the exposed region (ADER; Zeldin *et al.*, 2013 ▸) was calculated for 1 s exposure without rotation. This allowed the calculation of the cumulative dose at which each spectrum was collected. Thin films were modelled as crystals with a 100% solvent content and an average thickness of 40 µm, where the thickness does not significantly influence the calculation. See Supplementary Table S1 for details.

#### 2.3.2. Processing of UV–Vis absorption spectra

The collected UV–Vis absorption spectra under X-ray irradiation often show an achromatic increase in optical density (OD) over the UV–Vis range. Correction was made for this constant-baseline drift induced by the beam using the *in crystallo* optical spectroscopy (*ic*OS) toolbox (Caramello *et al.*, 2025 ▸). The average absorbance of the near-IR region (750–850 nm) was subtracted from each spectrum. Spectra were smoothed using a Savitzky–Golay filter (by fitting a third-degree polynomial over 21 consecutive data points). Dose constants τ were calculated by fitting mono-exponential curves to the dose-dependent change in absorption at a specific wavelength, using the equation
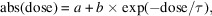
where *a* and *b* are positive constants. All spectroscopic figures were generated within the *ic*OS toolbox (Caramello *et al.*, 2025 ▸).

## 3. Results

### 3.1. Monitoring spectroscopic signatures upon X-rayirradiation

In this work, we establish protocols to monitor changes in UV–Vis absorbance in buffers or crystals upon X-ray irradiation. This is essential when studying chromophores within proteins, such as ligands or reaction intermediates. The task is complex, as a protein crystal in a flash-cooled loop contains the ligand bound to the protein, and the protein itself is prone to SRD. Furthermore, the complex is immersed in amorphous ice containing crystallization buffer components and cryoprotectant, all of which may respond to the incident X-ray radiation by changes in UV–Vis absorbance. Therefore, (all) individual components are to be tested to determine how they change over the duration of an X-ray data collection. The methods presented here are based on several experiments over the past years, debugging the protocols from obvious pitfalls.

These experiments require homogeneous samples with uniform X-ray illumination. This requirement restricts them to being carried out with top-hat beam profiles; we have used beamline BM07-FIP2 and, in earlier preliminary experiments, beamline BM30A-FIP at the ESRF. The requirement of producing homogenous samples is difficult to achieve, requiring the amorphous cooling of buffers in loops where the obtained films are of uniform thickness. We found that this could best be achieved by using MiTeGen loops, because of the way these are produced as stamps from a Kapton film of uniform thickness. Loops of 100 µm in diameter are generally preferred, as they lead to more even films than the larger 200 µm loops, and additionally their design with the wicking hole allows liquid to drain. The 100 µm loops were, however, more difficult to align with the spectrophotometer so that there was no inherent signal from the Kapton film.

**Figure 1:**
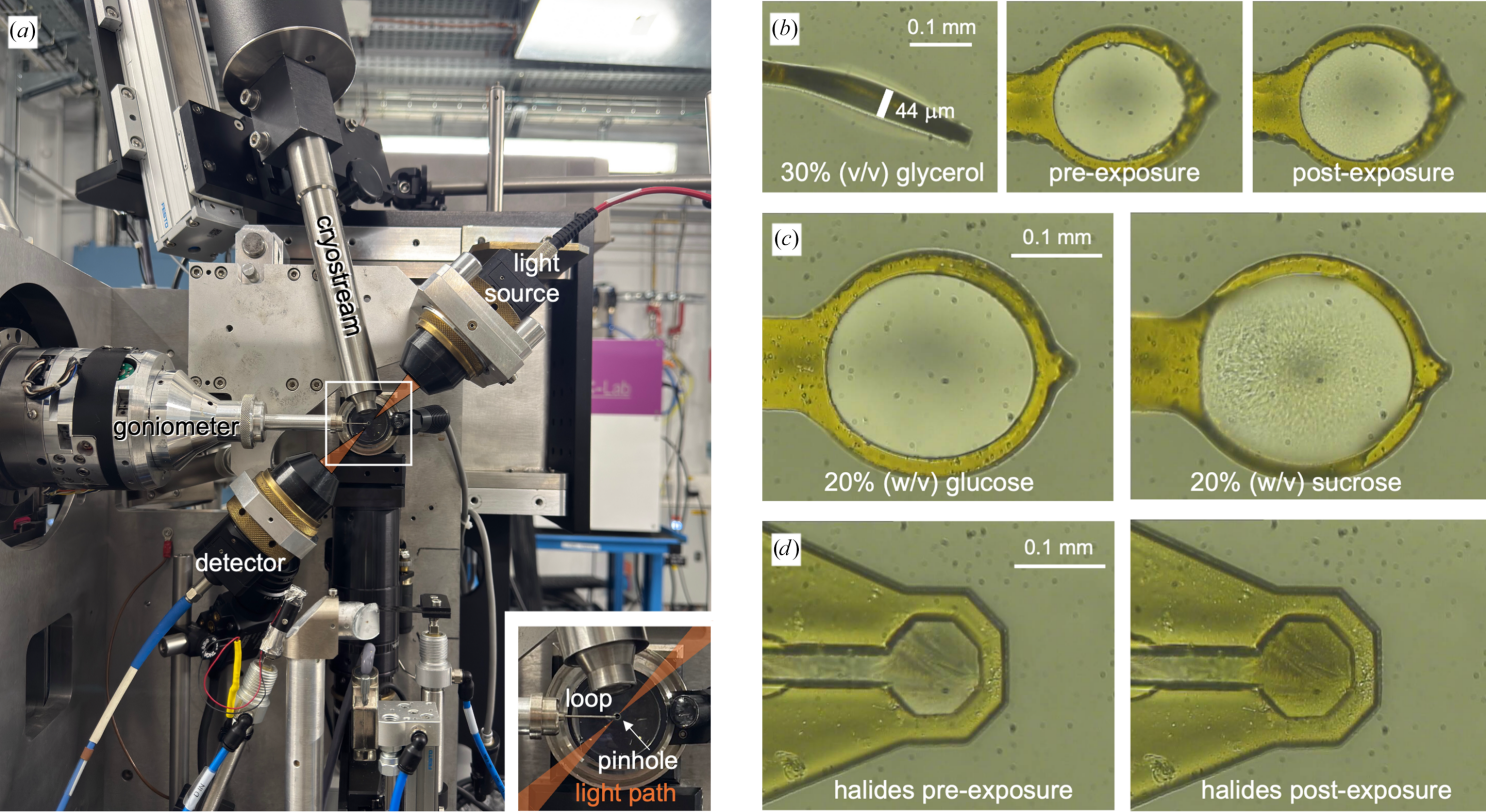
Experimental setup for UV–Vis absorption spectroscopy acquisition with examples. (*a*) The loop with the sample was mounted on the goniometer at ESRF beamline BM07-FIP2 in the cryostream and was not rotated during X-ray exposure; the UV–Vis absorption microspectrophotometer lamp and sensor were aligned with the sample. Inset: on-axis view with the top-hat X-ray beam (200 × 200 µm in all experiments) passing through the on-axis camera objective pinhole onto the mounted sample, with the light path orthogonal to the X-ray beam. (*b*) On-axis images of a mounted loop, here 30% glycerol. Left: thickness is determined with the loop edge-on. Right: the face-on view allows evaluation of the sample transparency, shown here before and after X-ray exposure. (*c*) Flash-cooling of 20%(*w*/*v*) glucose resulted in a transparent thin film, but 20%(*w*/*v*) sucrose did not, highlighting challenges in producing homogeneous vitreous films. (*d*) The Morpheus halide additive mixture is an example of a solution that did not lead to a transparent vitreous film upon flash-cooling and additionally showed visible changes after X-ray exposure.

The ESRF/EMBL microspectrophotometer (McGeehan *et al.*, 2009 ▸; von Stetten *et al.*, 2015 ▸) as set up on beamline BM07-FIP2 is shown in Fig. 1 ▸(*a*). Samples were mounted by dipping the MiTeGen loop into a droplet of sample, starting with 200 µm loops. To mount a sample, the base of the pin was placed onto the goniometer magnet at an angle of roughly 45°, and the pin was then quickly tilted into the aligned position within the cryostream. Samples were oriented perpendicular to the direction of the light path, and the thickness of the film was noted (Fig. 1 ▸*b*). The sample was checked visually to see whether a film of uniform thickness was achieved. For samples that showed high absorption across the spectrum, the mount was repeated, also trying smaller 100 µm loops to achieve thinner films.

Mounting of samples directly in the cryostream often resulted in non-amorphous films where the sample was not entirely transparent. The mounting was frequently repeated to achieve the most transparent films, rather than annealing the sample by blocking the cryostream. Fig. 1 ▸(*c*) demonstrates this effect, where transparent films were obtained with 20% glucose, but not with 20% sucrose. Samples that are not perfectly transparent due to the formation of small inhomogeneities would lead to Rayleigh scattering (Caramello *et al.*, 2025 ▸), which results in a loss of those photons scattering away from the light path, and thus an increase of the absorption towards shorter wavelength, as Rayleigh scattering inversely scales with the fourth power of the wavelength. This was checked by inspection of the spectrum, if necessary repeating the sample mounting to minimize this effect.

Furthermore, (some) transparency was often lost after X-ray exposure, as is evident from the images of the glycerol sample shown in Fig. 1 ▸(*b*). With some samples such as the halide additive mixture from the Morpheus screen (Gorrec, 2009 ▸) we were unable to produce clear films, and additionally observed a further loss of transparency with prolonged X-ray exposure (Fig. 1 ▸*d*), suggesting that samples scatter light before irradiation, and this would further increase during the experiment.

To perform experiments, the sample thickness and flux must first be determined, allowing the calculation of dose (see Section 2.3.1). The data in Section 3.1 were collected over two beamtimes, where the flux was determined using a silicon pin diode (Owen *et al.*, 2009 ▸) to be 3.5 × 10^11^ and 6.25 × 10^11^ photons s^−1^, with the ESRF operating at a ring current of 200 mA. For the measurements, the beam size was set to be larger than or to match the sample, while the sample itself was larger than the observation window of the microspectro­photometer. The X-ray beam size used on beamline BM07-FIP2 was 200 × 200 µm, while the focal spot of the microspectrophotometer had a diameter of 50 µm.

The spectrophotometer was set up to continuously collect either 500 ms or 1 s spectra between 280 and 800 nm, depending on the experiment. Ten spectra were collected prior to the opening of the X-ray shutter. During spectroscopic acquisition, the goniometer remained stationary. Whilst initial experiments involved the collection of a long series of spectra (over an hour), we observed that the majority of changes occurred rapidly. We thus limited the total data-collection time to 10 min, which incidentally also mimicked a typical data-collection scenario for a macromolecular crystal dataset on beamline BM07-FIP2 more closely. The strategy we employed for all experiments described here is summarized in Supplementary Fig. S1. In the following and in the supporting information, we give examples for various buffers typical in macromolecular crystallography.

#### 3.1.1. Crystallization buffer constituents

We investigated crystallization components individually, including common precipitants such as PEG and salts, as well as typical buffers. Early radiation-chemistry studies provided a framework for interpreting the observed spectral phenomena at specific peak maxima. For example, the dichlorine radical anion, 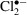, exhibits a characteristic absorption at λ_max_ = 340 nm in 2 *M* NaCl (Anbar & Thomas, 1964 ▸), the hydroxyl radical 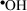 generated by water radiolysis absorbs at λ_max_ = 280 nm (Ershov & Pikaev, 1968 ▸), the superoxide radical anion 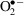 absorbs in the deep-UV region at λ_max_ = 245 nm (Janik & Tripathi, 2013 ▸) and solvated electrons give rise to a peak at λ_max_ = 560 nm in 40% glycerol (Ershov & Pikaev, 1968 ▸). Radiation chemistry has further revealed that glycerol undergoes conversion into a mixture of compounds, including malondialdehyde and glyoxylic acid, with absorption maxima at λ_max_ = 260  and 220 nm, respectively (Ivanova *et al.*, 2009 ▸; Eugene *et al.*, 2016 ▸). In addition, the concentration dependence of the solvated electron peak in glycerol has been documented (Ershov & Pikaev, 1968 ▸; McGeehan *et al.*, 2009 ▸).

**Figure 2:**
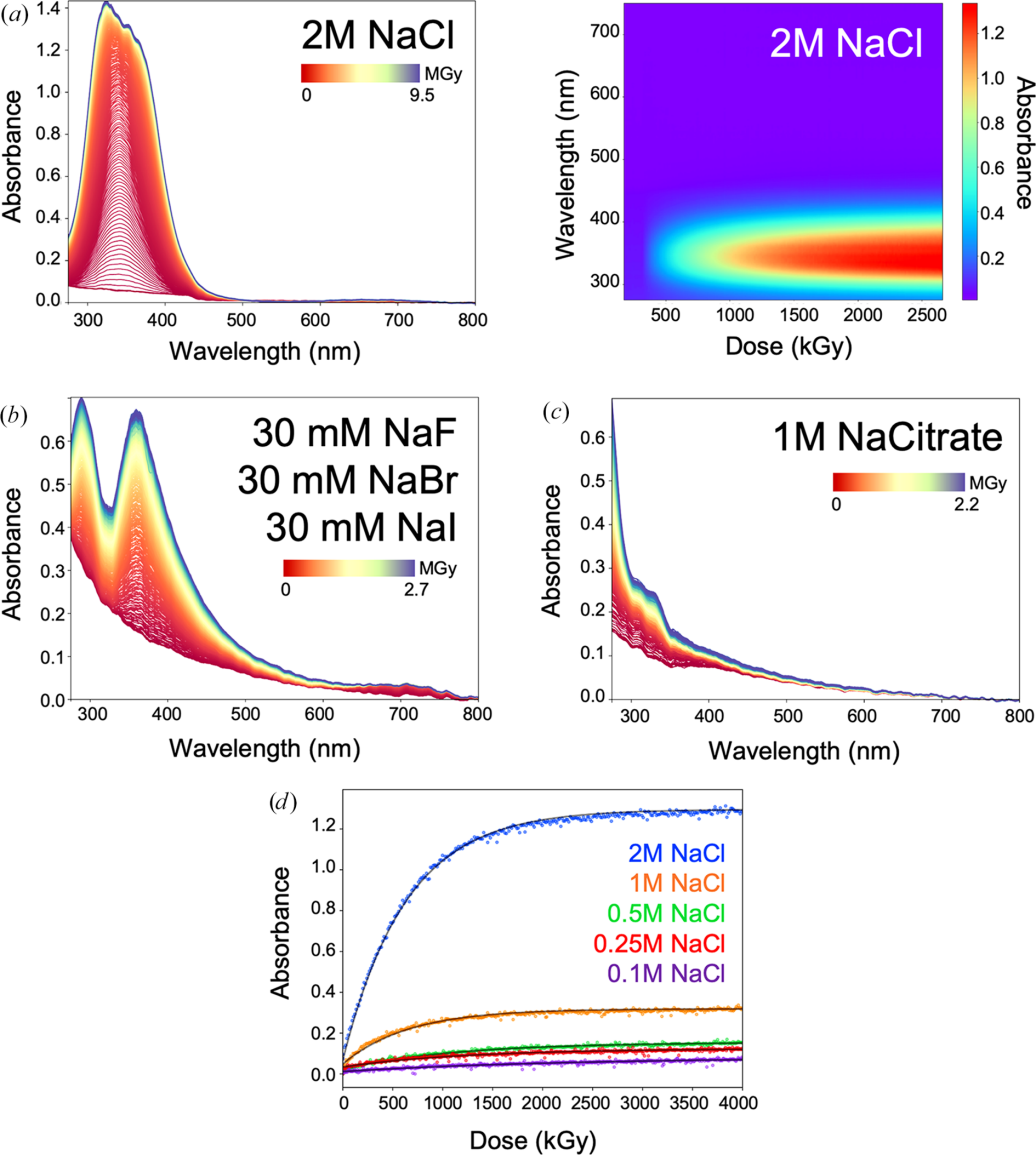
UV–Vis absorption spectroscopy changes induced by X-ray dose absorbed by sodium chloride and halogen substitutes. (*a*) UV–Vis absorption spectrum of 2 *M* sodium chloride under X-ray exposure, showing a distinct absorption peak at 345 nm. The change with increased dose is shown using a colour gradient from red at the start of the measurement to blue at the end of the measurement. The corresponding 3D plot underneath reveals the change with dose against increased absorption from blue (minimal change) to red (maximal change). (*b*) Substitution of the chloride anion in sodium salts with fluorine, bromine and iodine results in two distinct absorption peaks (λ_max_ = 290 and 360 nm). (*c*) Substitution of the chloride anion with citrate significantly reduces the spectroscopic response. (*d*) The dose-dependent rise in absorption at 345 nm of the NaCl sample plateaus at approximately 2000 kGy. Mono-exponential fits (black lines) derive dose constants of 0.63 MGy (2 *M* NaCl), 0.66 MGy (1 *M* NaCl), 1.44 MGy (0.5 *M* NaCl), 1.25 MGy (0.25 *M* NaCl) and 2.28 MGy (0.1 *M* NaCl).

We first experimentally tested several sodium salts. As expected from previous studies, sodium chloride shows a rather dramatic response, with a distinct absorption peak (λ_max_ = 345 nm) developing rapidly (Fig. 2 ▸*a*). This response is known to originate mainly from the dichlorine radical anion, 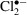 (Anbar & Thomas, 1964 ▸). We therefore tested other sodium halogen salts, for which we used the Morpheus halide additive mixture (Gorrec, 2009 ▸) consisting of sodium bromide, iodide and fluoride (Fig. 2 ▸*b*). Several absorption peaks are indicative of the halogens, similar to that which was observed with sodium chloride. The tri-iodide anion 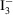 was previously shown to absorb at λ_max_ = 288 and 352 nm (Kireev & Shnyrev, 2015 ▸), matching the specific absorption peaks observed here. In contrast, sodium citrate shows a much less dramatic response compared with sodium chloride (Fig. 2 ▸*c*). In the halide and sodium citrate samples, an increase in absorption towards shorter wavelengths was observed, and this effect was already apparent in the non-irradiated state at the beginning of the dose series (red curves), consistent with light scattering. Unlike a distinct absorption peak, light scattering leads to gradual photon loss on the spectrophotometer detector, which reduces the signal-to-noise ratio of the measurement. Finally, sodium chloride exhibited a sample-concentration dependence that was inversely related to the dose constant at λ = 345 nm, with higher concentration solutions leading to a higher OD increase, and the signal plateauing at doses of ∼2 MGy (Fig. 2 ▸*d*). While the dose constant determined for 1 *M* sodium chloride was 0.66 MGy, the absorbance at λ = 345 nm did increase similarly rapidly for 1 *M* sodium citrate, with a dose constant of 0.88 MGy (not shown).

**Figure 3:**
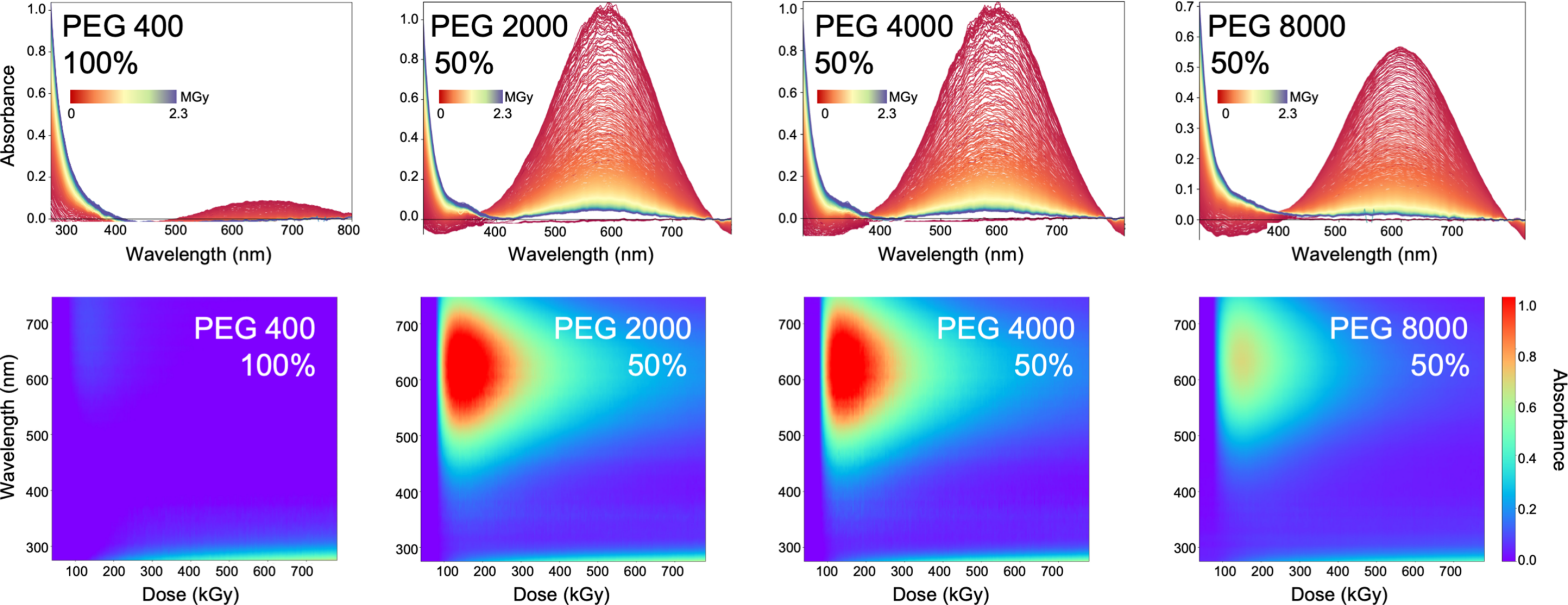
Polyethylene glycol (PEG) solutions of varying molecular weight exhibit a transient solvated electron peak in the 450–700 nm range. Peak intensity is influenced by film thickness. With increasing dose the peak diminishes, and a second absorption feature emerges below 400 nm, most prominently in the 100% PEG 400 sample. The concentration of samples is given as percentage (*v*/*v*). Colour coding for 2D spectra is from red (start) to blue (end). In the 3D spectra, colour coding represents absorbance change with dose from blue (minimal change) to red (maximal change).

PEG solutions of varying molecular weight are often used in crystallization. These produce a transient solvated electron peak in the 450–700 nm range (Fig. 3 ▸). PEG 400 was analysed ‘neat’ and showed an absorption peak at 610 nm, while 50% solutions of PEG 2000, PEG 4000 and PEG 8000 all had absorption maxima at 590 nm. Blue shifting of the solvated electron peak likely occurred as a concentration-dependent effect (McGeehan *et al.*, 2009 ▸). The different peak intensities for the higher molecular weight PEGs relate to the film thickness measured, which was 93, 100 and 63 µm for PEG 2000, PEG 4000 and PEG 8000, respectively (Supplementary Table S1). The peak resulting from solvated electrons disappears over time, while an increasing absorption is observed below 400 nm. This could have arisen either from increased scattering or from the formation of hydroxyl radicals (see Section 3).

**Figure 4:**
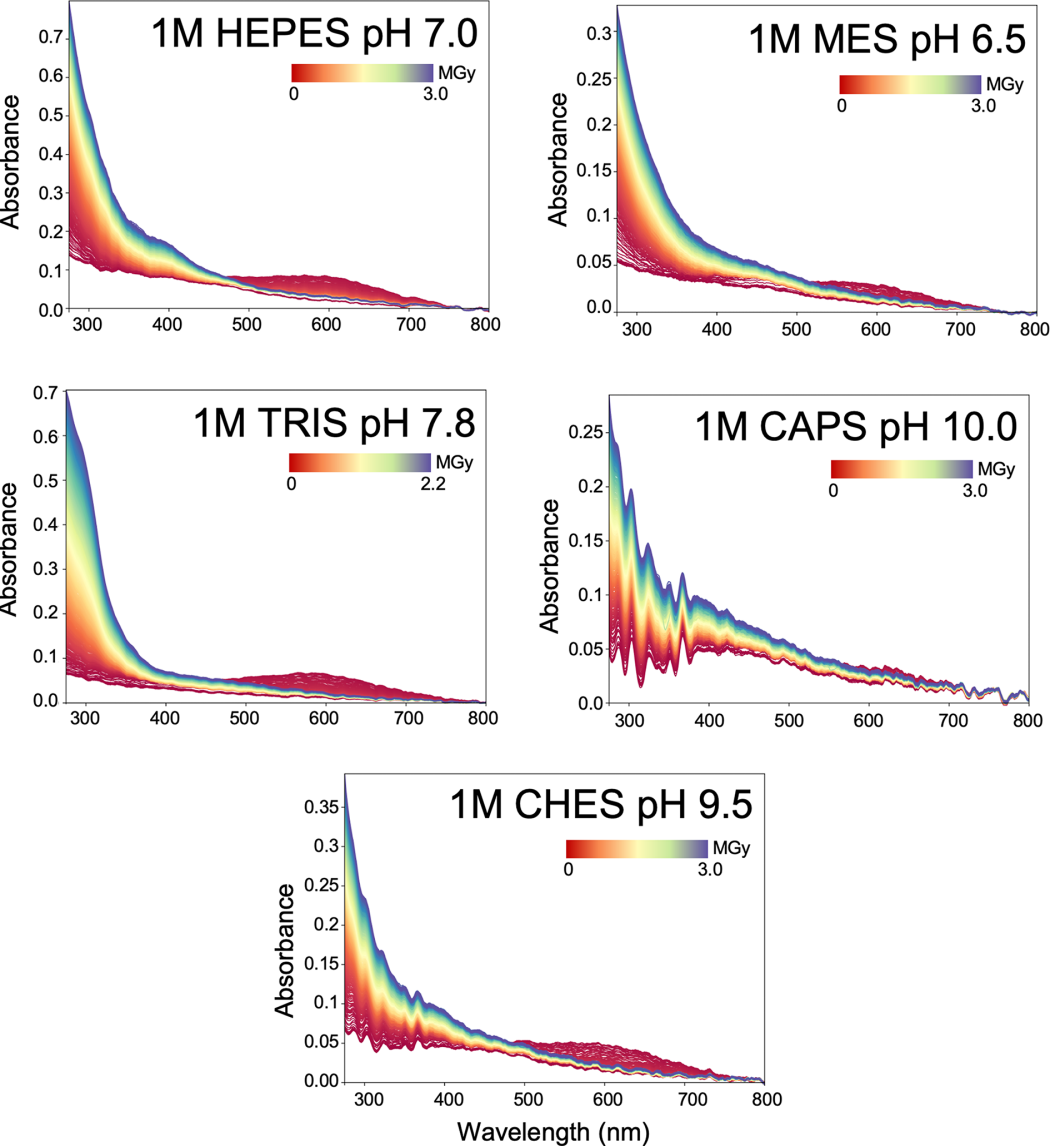
Effect of X-ray irradiation on common crystallization buffers. HEPES, MES, Tris, CAPS and CHES (all at 1 *M* concentration) produce a solvated electron peak near 600 nm, with CAPS buffer showing the weakest response. The spectra series are represented from red (start) to blue (end).

Typical crystallization buffers show prominent responses to X-ray irradiation. We compared HEPES, MES, Tris, CAPS and CHES, which all show a solvated electron peak at ∼600 nm, but this is weakest in the CAPS buffer (Fig. 4 ▸). Increasing absorption is observed below 400 nm, probably arising from increased scattering.

#### 3.1.2. Cryoprotectants

Glycerol, which is commonly used as a cryoprotectant, has been intensely studied in radiation chemistry. The presence of glycerol directly contributes to the solvated electron peak, with a concentration dependence for λ_max_ that shifts from 490 nm at 100% glycerol to ∼590 nm at 40% glycerol (Ershov & Pikaev, 1968 ▸; McGeehan *et al.*, 2009 ▸). The maximum at long wavelengths (500–600 nm) is a well documented feature caused by the absorption of light by solvated electrons generated by the photoelectric effect during X-ray irradiation.

The transient nature of the solvated electron peak is explained by the recombination of electrons with solvent molecules, leading to the formation of anionic radical species, where the rate of decay and extent to which the absorption returns to the baseline value is dependent on the solvent composition (Le Caër *et al.*, 2016 ▸). After the initial irradiation, increase of the peak at shorter wavelengths between 200 and 240 nm is observed. Reactions with solvated electrons also lead to changes at shorter wavelengths as observed previously in protein crystals (Owen *et al.*, 2012 ▸).

**Figure 5:**
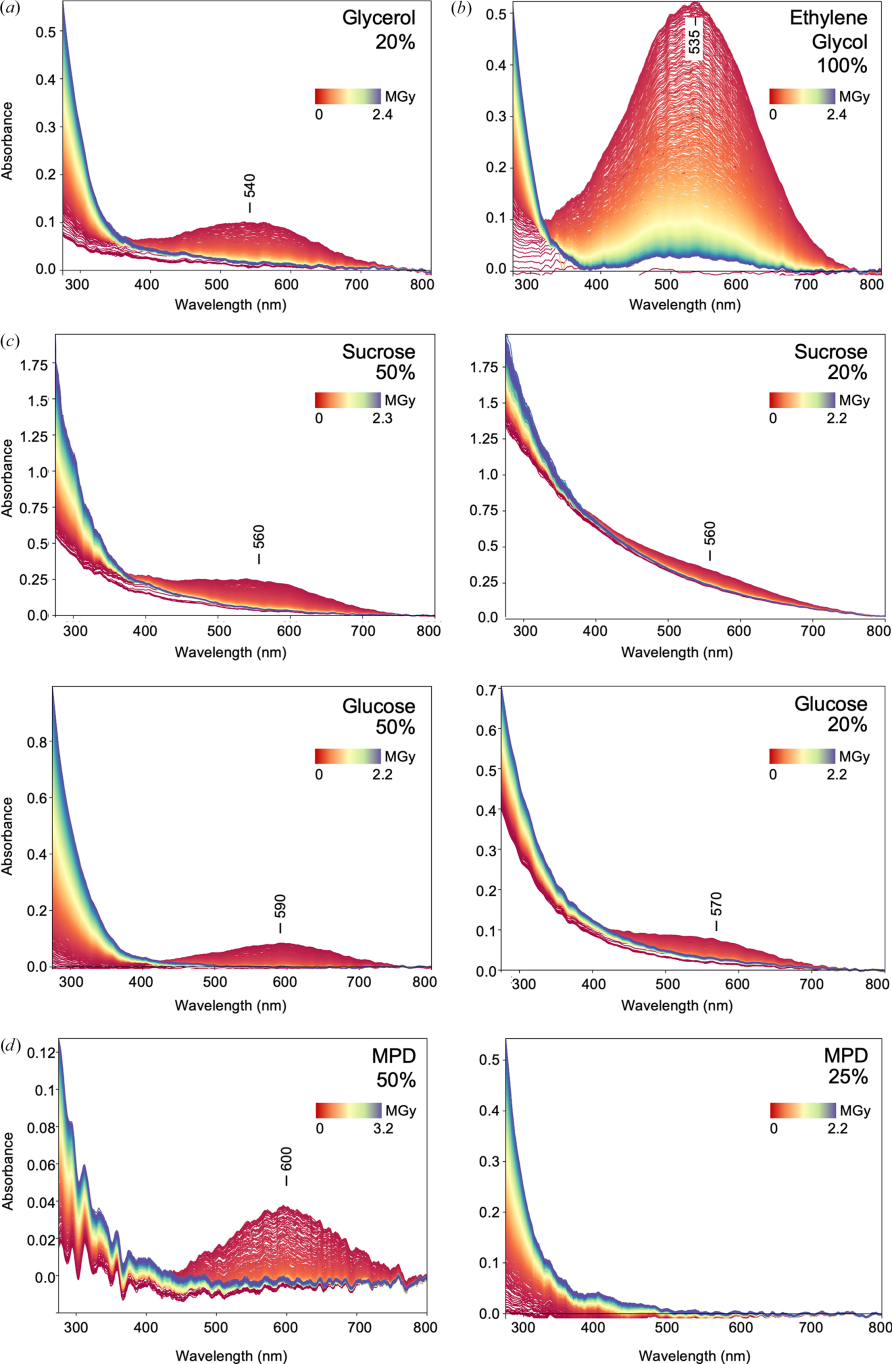
Effect of X-ray irradiation on commonly used cryoprotectants. (*a*) The distinct transient UV–Vis absorption peak at 540 nm arises from light-absorbing solvated electrons in 20% glycerol. (*b*) With 100% ethylene glycol, the solvated electron peak similarly appears with a maximum at 535 nm. (*c*) The solvated electron peak is less pronounced in saccharides such as glucose or sucrose, while the signal intensity reduces at lower concentrations of the saccharides, with maxima between 560 and 590 nm at the measured concentrations of 20% and 50%. (*d*) 2-Methyl-2,4-pentanediol (MPD) exhibits a transient peak near 600 nm at 50%, which is absent at 25%(*w*/*v*) concentration. The spectra series are represented from red (start) to blue (end).

We wanted to understand whether different cryoprotectants may be beneficial to reducing these effects and tested whether glycerol (Fig. 5 ▸*a*) could be replaced by ethylene glycol, saccharides or MPD. Ethylene glycol shows a strong response in the solvated electron peak region around 550 nm (Fig. 5 ▸*b*). The 600 nm peak is less pronounced for sucrose and even less so for glucose (Fig. 5 ▸*c*). Decreasing the concentration of the saccharide lowered the solvated electron peak; however, the samples did not lead to transparent films, as apparent from an increase in scatter (Figs. 1 ▸*c* and 5 ▸*c*).

We then tested MPD, which is often used as a crystallization precipitant but can also be used as a cryoprotectant. At a concentration of 50%(*v*/*v*) a transient peak in the solvated electron region is detected at around 600 nm, whilst it is almost absent at 25%(*v*/*v*) (Fig. 5 ▸*d*). There is, however, a gradual increase in absorption observed below 450 nm. Still, in comparison MPD may be a better choice of cryoprotectant for spectroscopic studies.

#### 3.1.3. Complex mixtures: SRD in the Morpheus crystallization screen

Conceivably, the various components of a cryoprotected crystallization mixture may influence each other during crystallographic data collection. We tested this using the Morpheus screen (Gorrec, 2009 ▸), which is not only widely used but has the advantage that it contains cryoprotectant in all crystallization conditions, allowing the user to directly transfer and flash-cool the crystal.

We tested how the mixtures in the Morpheus screen behaved (Gorrec, 2009 ▸). The precipitant mixtures P1–P4 showed similar behaviour to the individual components (Supplementary Fig. S2, compare Figs. 3 ▸, 5 ▸*a*, 5 ▸*b* and 5 ▸*d*). However, we recorded distinctive signatures for buffer and additive mixtures. Buffer system 1 (BS1) showed an increase of the baseline towards shorter wavelengths that increased during X-ray exposure (Supplementary Fig. S3), strongly indicative of light scattering, which ties in with difficulties in obtaining a transparent film for this sample in the cryostream. Buffer systems 2 (BS2) and 3 (BS3) were easier to prepare as film samples, and consequently also showed less scattering in the non-X-ray-exposed samples; however, these samples also showed a buildup towards shorter wavelengths during X-ray exposure, which again is attributed to loss of photons as they scatter away from the light path. BS2 produces the transient solvated electron peak at ∼600 nm. For completeness, we also document the effects obtained for the Morpheus additive mixtures (Supplementary Fig. S4), which are mostly scattering effects, though the ethylene glycol mixture showed a solvated electron peak (compare Fig. 5 ▸*b*), while the halogens mixture produced discrete peaks as shown in Fig. 2 ▸(*b*).

### 3.2. Mapping buffer effects with proteins to monitor SRD

This study was motivated by an earlier observation made during the study of reaction intermediates in vitamin B_6_ biosynthesis. We previously published a structural investigation of the chromophore I320 in the Pdx1 enzyme (Rodrigues *et al.*, 2017 ▸). The five-carbon adduct forms as a double Schiff base between two catalytic lysine residues. Resolving the structure of this key covalent intermediate led to a novel interpretation of the catalytic pathway, proposing a handover between two Schiff-base-forming lysines in a previously unanticipated use of imine chemistry (Robinson *et al.*, 2016 ▸; Rodrigues *et al.*, 2017 ▸). As Schiff bases are often radiation-sensitive (Giardina *et al.*, 2017 ▸; Dubnovitsky *et al.*, 2005 ▸), we wanted to use spectroscopy to confirm its structure and avoid misinterpretation of the electron density.

**Figure 6:**
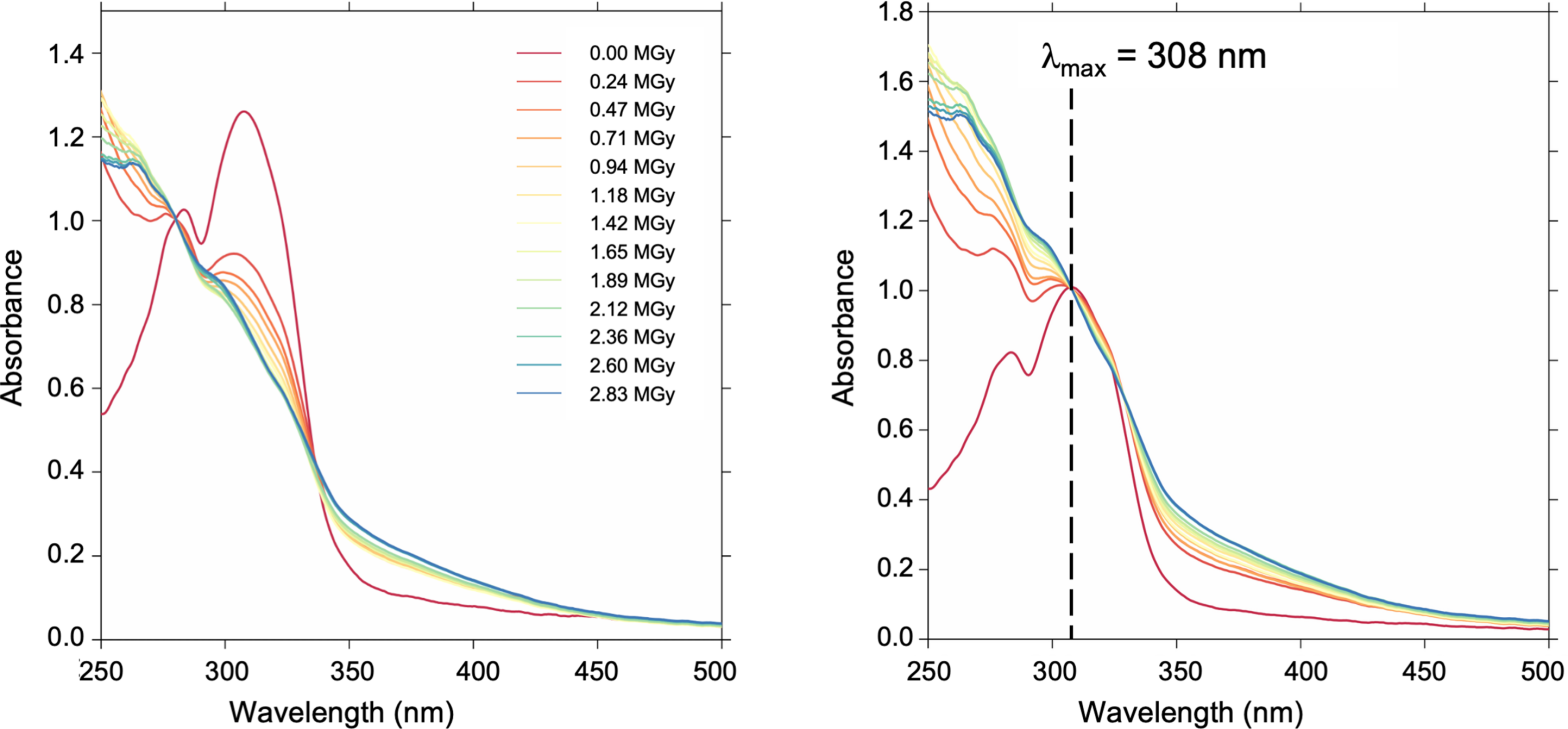
Monitoring specific radiation damage (SRD) in the Pdx1–I320 intermediate complex. Continuous UV–Vis absorption spectroscopy data collection up to 2.8 MGy suggests that the peak for the intermediate Pdx1–I320 decreases with continued X-ray exposure (left-hand panel). Normalizing to the intermediate peak (λ_max_ = 308 nm) reveals degradation of the signal due to an increase of background/loss of photons in the deep-UV part of the spectrum (right-hand panel). Colour coding is indicated in the left-hand panel.

While the initial experiments were carried out a decade ago on ESRF beamline ID14-4, we repeated this experiment on beamline BM07-FIP2 with the current setup. Continuous collection of UV–Vis absorption spectra up to 2.8 MGy showed a waning of the I320 signal (Fig. 6 ▸, left-hand panel). As we could not be certain that the intermediate was undamaged, we resorted to a multi-crystal low-dose data-collection strategy. To our surprise, we were able to confirm the structure and found that the electron density of the 245 kGy low-dose structure (PDB entry 5lnv) and that of the high-resolution single-crystal dataset determined at a dose of ∼1.5 MGy (PDB entry 5lnu) were virtually identical (Rodrigues *et al.*, 2017 ▸).

From the studies provided here it is now evident that buffer effects masked the I320 signal. Contributions arise from the crystallization components sodium citrate (550 m*M*) and HEPES (pH 7.0, 100 m*M*), as well as from the added cryoprotectant (20% glycerol) (Figs. 2 ▸*c*, 4 ▸ and 5 ▸*a*). The increase of absorption at shorter wavelength could conceivably arise from increased scattering, leading to a loss of photons in the deep-UV region, best visualized by normalizing for the intermediate peak at λ_max_ = 308 nm (Fig. 6 ▸, right-hand panel). Thus, both buffer absorption and scattering effects strongly overlap with signals below 400 nm and cannot be neglected in the study of chromophores, highlighting the importance of reliable protocols to detect these changes upon X-ray radiation during data collection.

## 4. Discussion

The assignment of radiation-damage effects when studying protein ligands can be complex. A useful approach is online *in crystallo* UV–Vis absorption spectroscopy (Gotthard *et al.*, 2019 ▸; Caramello & Royant, 2024 ▸). This technique enables the study of typical enzymatic ligands such as hemes, flavins or metals. Recording an initial spectrum allows verification of the specific state of the ligand before data collection. A change in absorption after the start of data collection would be indicative of a structural change in the ligand that should be attributed to a modification of its chemical structure induced by the incident X-ray irradiation. However, buffer components, present in high concentration in the crystal, are typically also radiation-sensitive and can shadow the signal of the chromophore that is to be investigated.

A well known effect is the increase in solvated electrons that lead to increased absorption around 550 nm. Glycerol is a commonly used cryoprotectant, but glycerol radiolysis is also a major contributor to spectroscopic changes (Fig. 5 ▸*a*). Other potential cryoprotectants, such as low-molecular-weight PEG solutions, ethylene glycol or carbohydrates, show similar effects (Figs. 3 ▸, 5 ▸*b* and 5 ▸*c*). If MPD is used instead, the effects of radiolysis are much less pronounced in the spectra (Fig. 5 ▸*d*). Indeed, both MPD and carbohydrates have been investigated previously for their efficiency in radical scavenging in various crystallization agents (Allan *et al.*, 2013 ▸).

We document the transient nature of solvated electrons with commonly known hydrophobic precipitants, such as polyethylene glycol mixtures of varying molecular weight. The solvated electron features are typically transient and mostly disappear after 300–500 kGy, which is significant as this dose range is typical for a low-dose data-collection scenario at 100 K (Fig. 3 ▸). We also explored these as mixtures in crystallization formulations (Morpheus screen; Gorrec, 2009 ▸; see the supporting information). The documented changes in absorption at shorter wavelengths may complicate the analysis of chromophore radiation damage.

We describe several changes in wavelength ranges below 500 nm that are characteristic of radiolysis of buffer components and therefore persist throughout data collection. We observe concentration-dependent effects with sodium chloride in the region below 400 nm that still require mechanistic explanation (Fig. 2 ▸*a*). In comparison, sodium citrate shows a much reduced response, implying that the changes seen with sodium chloride are due to the halide anion (Anbar & Thomas, 1964 ▸). Testing a halide sodium salt mixture confirmed the rise of distinct absorption peaks (Fig. 2 ▸*b*). Choosing an appropriate salt and buffer system (Fig. 4 ▸ and Supplementary Fig. S3) when performing UV–Vis absorption spectroscopy is therefore advisable.

X-ray irradiation will also affect proteins directly, resulting in specific radiation damage (SRD). For example, pulse radiolysis experiments with cysteine, glutathione and penicillamine have identified that X-ray irradiation-induced radicalization of thiol groups can cause the formation of an absorption peak with λ_max_ between 310 and 350 nm (Hoffman & Hayon, 1973 ▸; Purdie *et al.*, 1971 ▸; Quintiliani *et al.*, 1977 ▸). The Pdx1 protein contains five free cysteine residues which would be prone to thiol radicalization. In the analysis of the Pdx1–I320 intermediate we therefore considered and detected SRD to cysteine side chains when we retrospectively analysed the earlier dose-series experiment (Rodrigues *et al.*, 2017 ▸). We observed negative difference density around the S atoms of the cysteine residues, demonstrating that the *F*_o*n*_ − *F*_o1_ maps were showing specific radiation damage. Detecting spectral changes from SRD is however shadowed under experimental conditions, where the evolution of spectra is dominated by buffer effects and in particular by light scattering.

Of note, there are two potential light-scattering events that are limitations of spectroscopic studies. It was previously reported that when collecting spectra from crystals one must correct for scattering at the crystal–solvent–air interface (Dworkowski *et al.*, 2015 ▸; Rodrigues *et al.*, 2022 ▸). The perpendicular orientation of the film limits but does not prevent these effects, and although they are therefore manageable, scattering by the sample is different and cannot easily be avoided. In some cases, we could not achieve transparent samples (Figs. 1 ▸*c* and 1 ▸*d*), and the effects of increased light scattering of the sample caused by X-ray irradiation can only be controlled by paying attention to the selection of buffers that limit these effects.

Our study demonstrates that the correction of these effects in UV–Vis absorption spectroscopy will be experimentally challenging at shorter wavelengths. The increase of light scattering during crystallographic data collection results in a loss of photons from the optical path of the spectrophoto­meter, meaning that the number of photons containing information is lowered. This makes observation of absorption peaks difficult as the signal disappears into noise. We demonstrate for the Pdx1 I320 complex that a strong signal from the chromophore ‘disappears’ completely as it is shadowed by buffer and scattering effects.

Further development and better analysis would be needed to truly deconvolute all effects from the spectroscopic signal. The goal would be to produce reference spectra for each component contributing, separating out solvent radiolysis and SRD of the chromophore and the protein. While correction is unlikely to be possible for signals towards the UV region, as the number of detected photons steadily decreases with dose, the features arising from solvated electrons in the 450–700 nm range could be quantified and then deconvoluted from SRD of the sample. It may be possible to fit and subtract the individual and varying peak in each spectrum across the series (intensity, wavelength maximum, peak width). This would allow the effective use of UV–Vis absorption spectroscopy to track and quantify SRD towards longer wavelengths.

The present study documents several typical effects that are encountered which can clarify spectroscopic changes upon X-ray irradiation. Using the *ic*OS toolbox (Caramello *et al.*, 2025 ▸), we demonstrate how to characterize these effects for buffer components. The motivation for understanding the radiation-sensitivity of a reaction intermediate is critical for the interpretation of its electron density. Understanding the electronic structure of reaction intermediates is a vital step in understanding catalysis. Conducting systematic control experiments underlying X-ray-induced spectroscopic changes in protein crystals is essential for any study aiming to use UV–Vis spectroscopy to monitor specific radiation damage.

## Supplementary Material

Supplementary Table and Figures. DOI: 10.1107/S2059798326002743/gm5122sup1.pdf
