# Supplementary material for: Mapping the effects of specific radiation damage and solvent radiolysis in buffers and crystals with online UV–Vis absorption spectroscopy
Source: Acta Crystallogr D Struct Biol. 2026 Apr 21;82(Pt 5):504–15. doi: 10.1107/S2059798326002743 (PMC13134000; doi:10.1107/S2059798326002743)
Supplement: Supplementary file 1 [file d-82-00504-sup1.pdf]

STRUCTURAL
BIOLOGY

Volume 82 (2026)

Supporting information for article:

Mapping the effects of specific radiation damage and solvent radiolysis in buffers and crystals with online UV–Vis absorption spectroscopy

Jack Stubbs, Nicolas Caramello, Matthew Rodrigues, Sylvain Engilberge, Eric Mathieu, Samuel L. Rose, Gwyndaf Evans, Antoine Royant and Ivo Tews

Figure	Sample	scan # for t = 0	Integration time (ms)	Ring current start (mA)	Ring current end (mA)	Calculated flux (ph/s)*10^{11}	Dose (kGy/s)	Thick-ness (μm)	Loop size (μm)
2A, 2D	2M NaCl	13	500	196.47	194.4	6.25	13.3	41	100
2D	1M NaCl	8	1000	198.53	195.84	6.25	10.2	25	100
2D	0.5M NaCl	7	1000	193.58	191.2	6.25	8.6	38	100
2D	0.25M NaCl	7	1000	190.45	199.17	6.25	7.8	40	100
2D	0.1M NaCl	7	1000	196.63	194.52	6.25	7.3	22	100
2B	Morpheus Halides (1:10)	10	500	199.72	198.49	3.55	4.6	20	100
2C	1M Sodium Citrate pH 4.75	10	500	195.75	194.47	3.39	3.8	28	100
3	100% PEG 400	10	500	198.62	197.59	3.52	3.9	91	200
3	50% PEG 2000	10	500	196.35	194.83	3.48	3.9	93	200
3	50% PEG 4000	10	500	194.23	200.32	3.51	3.9	100	200
3	50% PEG8000	10	500	199.87	198.62	3.47	3.9	64	200
4	1M HEPES pH 7.0	10	500	195.75	194.53	3.48	5.0	30	100
4	1M MES pH 6.5	10	500	200.25	199.03	3.56	5.1	30	100
4	1M TRIS pH7.8	10	500	194.86	193.68	3.41	3.8	24	100
4	1M CAPS pH 10	10	500	197.57	196.57	3.50	5.1	20	100
4	1M CHES pH 9.5	10	500	195.05	193.68	3.45	5.0	22	100
5A	20% glycerol	10	500	200.07	198.65	3.50	3.9	62	200
5B	100% EG	10	500	193.11	200.00	3.57	4.0	60	200
5C	50% Sucrose	10	500	198.08	196.80	3.50	3.9	78	200
5C	50% Glucose	10	500	195.84	194.60	3.46	3.8	50	200
5C	20% sucrose	10	500	197.5	196.21	3.42	3.8	44	200
5C	20% glucose	10	500	195.57	194.3	3.39	3.8	48	200
5D	50% MPD	10	500	195.52	194.31	3.45	3.8	32	200
5D	25% MPD	10	500	193.32	199.45	3.48	3.8	40	200
S2	Morpheus Precipitant Mix 1	10	500	199.63	198.42	3.45	3.8	75	200
S2	Morpheus Precipitant Mix 2	10	500	199.53	198.29	3.61	4.0	97	200
S2	Morpheus Precipitant Mix 3	10	500	197.26	196.1	3.60	4.0	95	200
S2	Morpheus Precipitant Mix 4	10	500	194.32	200.6	3.59	4.0	75	200
S3	Morpheus BS1	10	500	198.47	197.14	3.53	3.9	29	100
S3	Morpheus BS2	10	500	196.62	195.96	3.50	6.3	28	100
S3	Morpheus BS3	10	500	194.45	200.65	3.53	3.9	25	100
S4	Morpheus Carboxylic Acids	10	500	194.19	200.09	2.80	3.9	25	100
S4	Morpheus Monosaccharides	10	500	197.71	196.5	3.52	3.9	20	100
S4	Morpheus Ethylene Glycols	10	500	195.37	194.21	3.38	3.7	30	100
S4	Morpheus Amino Acids	10	500	197.70	196.52	3.42	3.8	40	100
S4	Morpheus NPS	10	500	198.76	197.55	3.54	4.7	28	100
S4	Morpheus Alcohols	10	500	196.41	195.26	3.49	3.9	23	100

Table S1. Experimental parameters for all samples collected during this study. The run was started by continuously collecting spectra, where t=0 denotes the first scan where the safety shutter was opened, representing the first spectra collected during X-ray exposure. All experiments used MīTeGen loops (Ithaca, NY, USA).

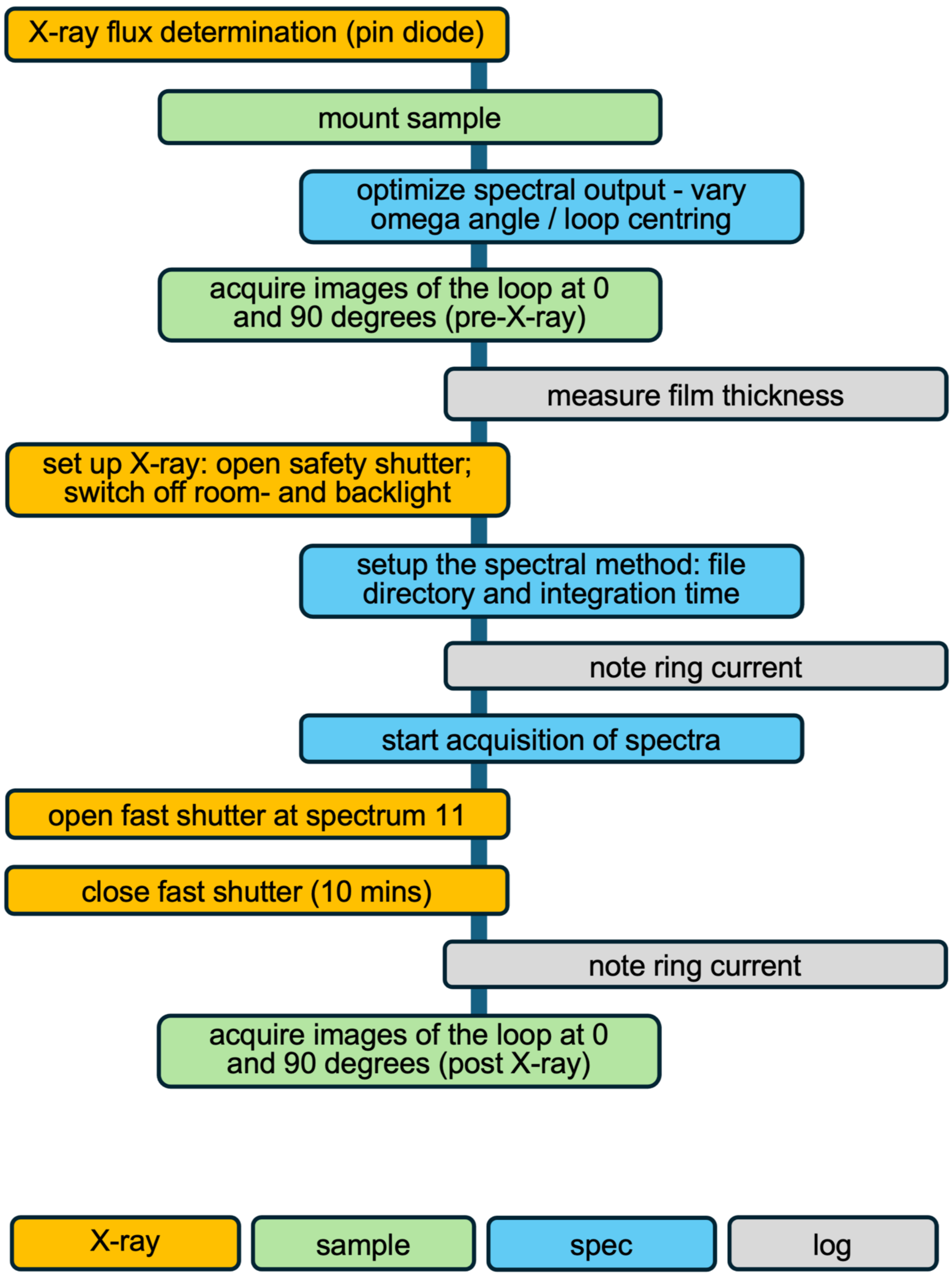

Figure S1. Workflow, listed for operations on the X-ray, sample, spectrophotometer, and log entries.

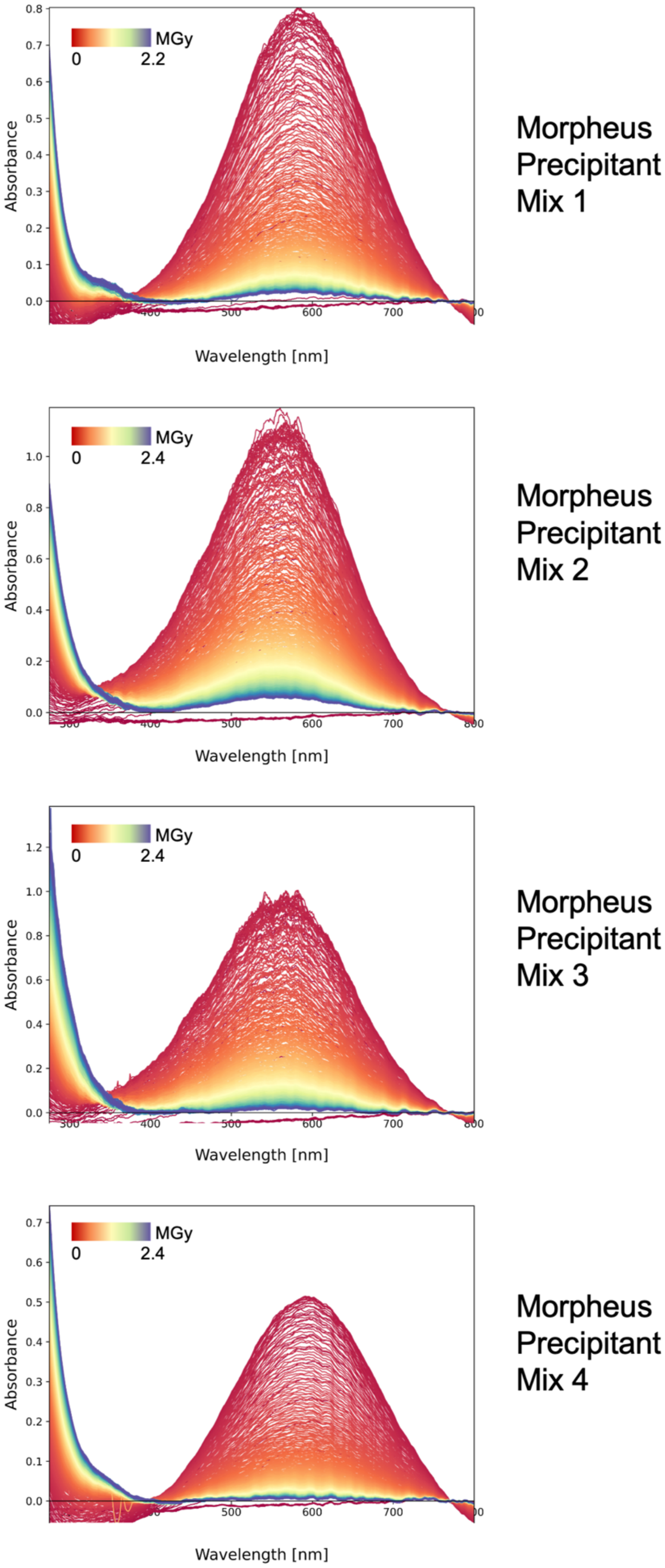

Figure S2. Morpheus ® Precipitant Mixes P1 (40% v/v PEG 500 MME and 20% w/v PEG 20,000), P2 (40% v/v ethylene glycol and 20% PEG 8000), P3 (40% v/v glycerol and 20% PEG 4000) and P4 (25% v/v MPD, 25 % w/v PEG 1000 and 25% w/v PEG 3350).

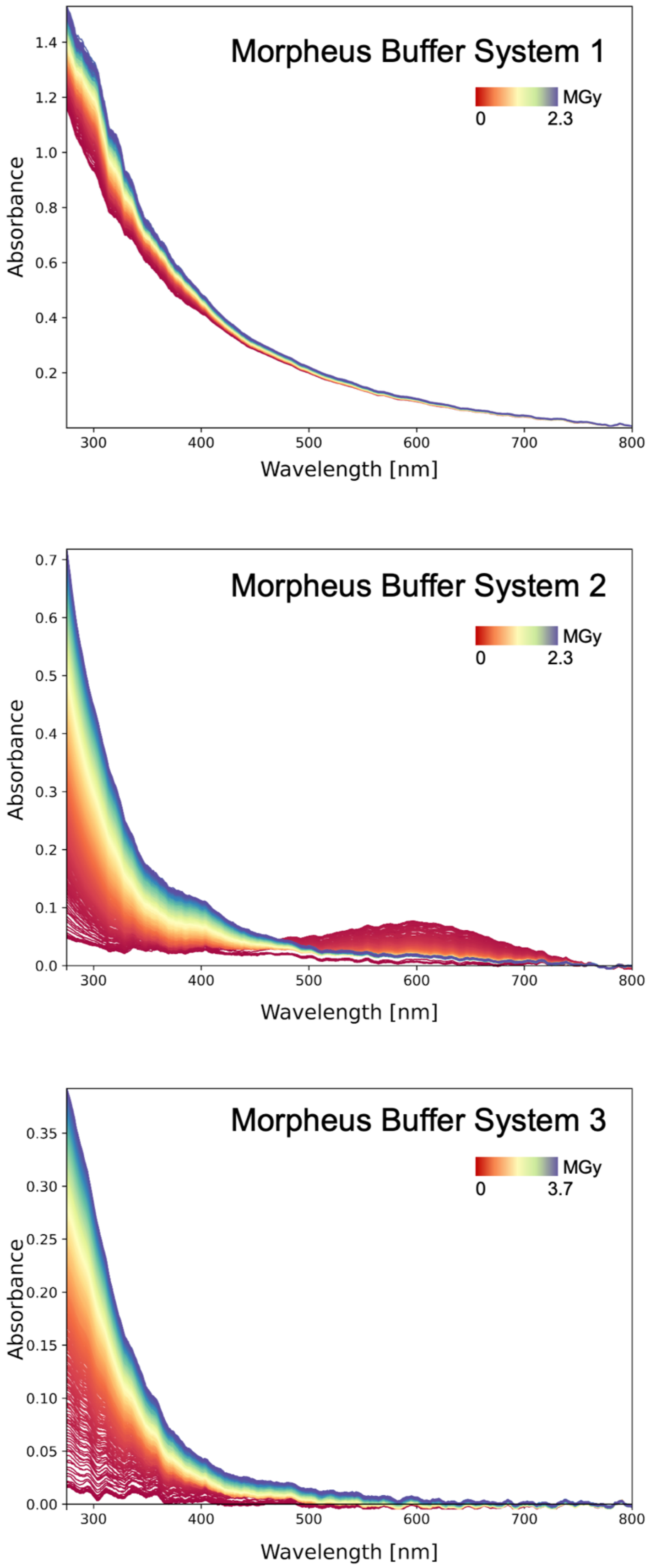

Figure S3. Morpheus ® Buffer System BS1 (imidazole/MES), BS2 (HEPES/MOPS) and BS3 (Tris/Bicine) all at 1M concentration.

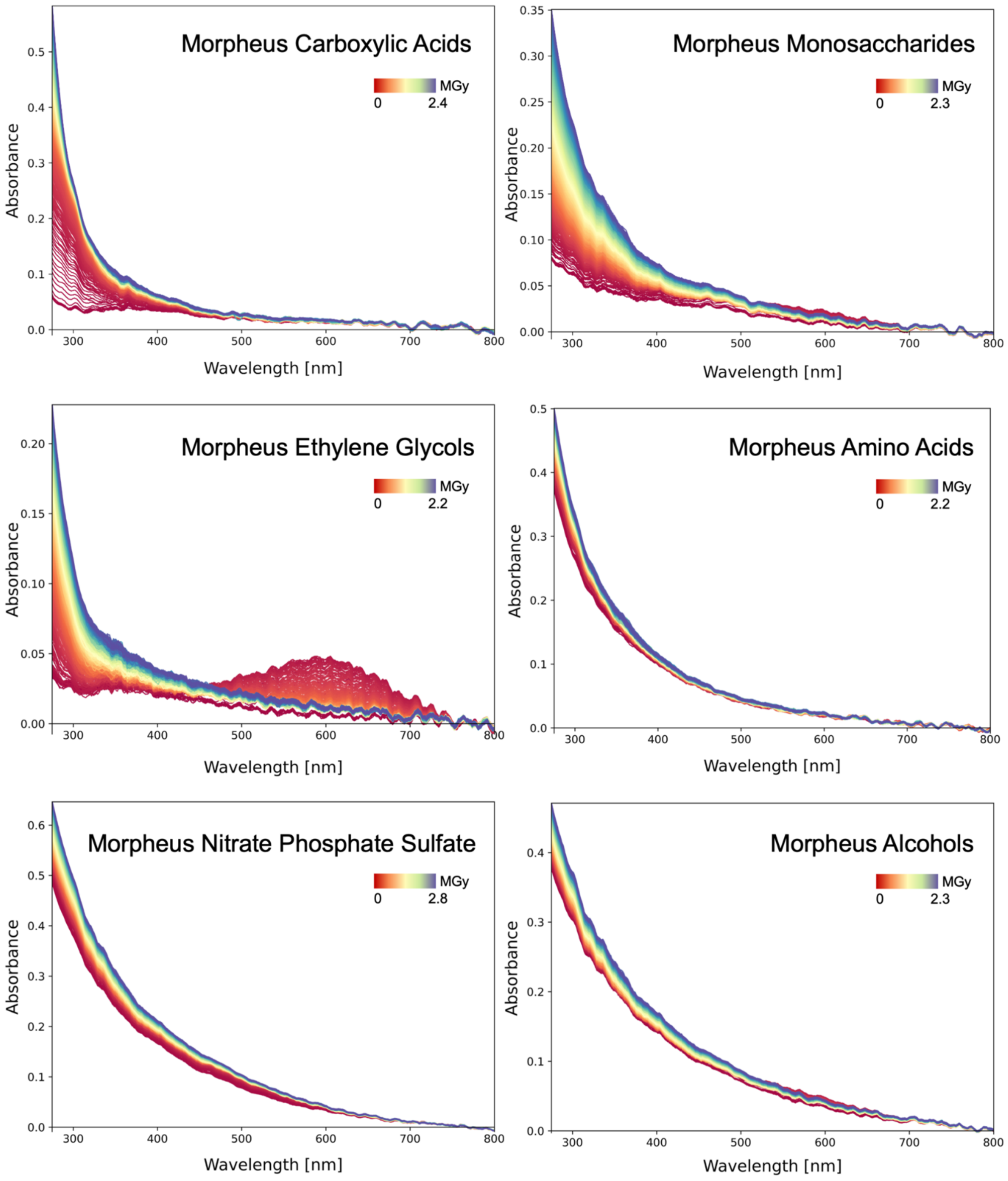

Figure S4. Selected additives from Morpheus (Gorrec, 2009) crystallization screen.
